# Electricity in the Diagnosis and Treatment of Diseases of the Nose, Throat, and Ear

**Published:** 1899-06

**Authors:** 


Electricity in the Diagnosis and Treatment of Diseases of the Nose, Throat, and Ear. By W. Scheppegrell, A. M., M. D., ex-Vice-President of the American Laryngological, Rhinological and Otological Society; late Assistant Surgeon to the Eye, Ear, Throat and Nose Hospital, New Orleans, etc., etc., New Orleans, La. Published by G. P. Putnam's Sons, New York, London. 1898. 381 pages. Price, cloth, $4.50.

This work is unique, in that no work confined to this subject has heretofore been published; and has the rare distinction of having been written by a southern physician. The writer is not able to criticise its merits, not being a specialist in these branches, and not being too familiar with the literature and therapeutics of electricity, but must say that it bears evidence of extensive research and thorough clinical experience on every page. The contents are divided into thirty-seven chapters, the last two being devoted to skiagraphy. In addition to this, is added an extensive bibliography, and list of authors. Dr. Scheppegrell is known by reputation to us all, and those interested in the subject should add his work to their libraries. We have no doubt that a more thorough knowledge of the therapeutic uses of electricity would be of advantage to us all. In this connection the author says: "A physician ignorant of the elements of electricity and its application in pathologic conditions, resembles some of the older practitioners, who, from their inability to use it refuse to admit the utility of the stethoscope, and who, in consequence, fail to take advantage of this means of diagnosis with which modern discovery has provided them." J.

